# Occlusal rehabilitation in patients with congenitally missing teeth—dental implants, conventional prosthetics, tooth autotransplants, and preservation of deciduous teeth—a systematic review

**DOI:** 10.1186/s40729-015-0025-z

**Published:** 2015-11-18

**Authors:** Hendrik Terheyden, Falk Wüsthoff

**Affiliations:** 1 Department of Oral and Maxillofacial Surgery, Red Cross Hospital, Hansteinstr. 29, D-34121 Kassel, Germany; 2 Department of Oral and Maxillofacial Surgery, Schleswig-Holstein University Hospital, Arnold-Heller-Straße 3, Haus 26, 2D-4105 Kiel, Germany

**Keywords:** Hypodontia, Oligodontia, Anodontia, Tooth aplasia, Tooth agenesis, Congenitally missing teeth, Tooth autotransplantation, Deciduous tooth, Dental implants, Ectodermal dysplasia

## Abstract

**Background:**

Implant patients with congenitally missing teeth share some common charateristics and deserve special attention.

**Methods:**

The PICO question was “In patients with congenitally missing teeth, does an early occlusal rehabilitation with dental implants in comparison to tooth autotransplants, conventional prosthetics on teeth or preservation of deciduous teeth have better general outcomes in terms of survival, success and better patient centered outcomes in terms of quality of life, self-esteem, satisfaction, chewing function?”

After electronic database search, a total of 63 relevant studies were eligible, of which 42 qualified for numerical data synthesis, 26 being retrospective studies. A data synthesis was performed by weighted means for survival/success/annual failure rates.

**Results:**

The mean survival of implants was 95.3 % (prosthesis survival 97.8 %), autotransplants 94.4 %, deciduous teeth 89.6 %, and conventional prostheses 60.2 %. The implant survival in children, adolescents, and adults was 72.4, 93.0, and 97.4 %. Annual failure rates of implants 3.317 %, autotransplants 1.061 %, deciduous teeth 0.908 %, and conventional prostheses 5.144 % indicated better results for natural teeth and more maintenance needs for the both prosthetic treatments. The mean OHIP score was 27.8 at baseline and a mean improvement of 14.9 score points was reported after implant prosthetics. The mean satisfaction rates were 93.4 (implants), 76.6 (conventional prostheses), 72.0 (autotransplants), and 65.5 % (orthodontic space closure).

**Conclusions:**

In synopsis of general and patient-centered outcomes, implants yielded the best results, however, not in children <13 years. Autotransplants and deciduous teeth had low annual failure rates and are appropriate treatments in children and adolescents at low costs. Conventional prosthetics had lower survival/success rates than the other options. Due to heterogeneity and low number of studies, patient-reported outcomes in this review have to be interpreted with caution.

## Background

Congenitally missing teeth, also called hypodontia, is the most frequent human malformation. The prevalence of hypodontia in white populations is estimated to be 5.5 %, with a higher incidence in women than in men. Hypodontia varies in severity, from a single missing tooth to the absence of all permanent teeth called anodontia. Oligodontia is usually defined as the absence of 6 or more permanent teeth, the third molars excluded, and its prevalence is estimated at 0.14 % in the white population [1]. Hypodontia can occur in isolation (non-syndromal) or as a part of numerous inherited syndromes of which the different forms of ectodermal dysplasia are the predominant entity [2]. Patients with congenitally missing teeth comprise a special group of patients deserving special attention, especially when dental implants are needed.

In cases with congenitally missing teeth, the defect in the dentition occurs very early in life, in contrast to many other implant patients who lost their teeth due to caries or periodontitis at later stages. The early time point has an advantage that the young patients are usually well adapted to the defects. However, prosthetic treatments are often necessary already in childhood. In childhood and adolescence, prosthetic treatments can be complicated, because teeth should not be ground as abutments for crowns due to the large pulp cavity, and dentures may not be splinted if the jaws still grow. It is also questionable whether dental implants can be placed before termination of growth due to the well-known problems of secondary infraocclusion due to the ankylotic healing of osseointegrated implants and due to other biological reasons [3]. Furthermore, children and their young parents and families often have a cost problem, since unlike other implant patient groups, the tooth defects appear in early phases of life when the income is low or needed elsewhere. In some public health systems, occlusal rehabilitation in childhood and adolescence is covered by public insurances and has to be finished before the 18th year of life.

The local implant site can be special, too. A site with aplasia of the primary or secondary tooth usually is different from implant sites in conventional implant patients. Usually, there is a severe lack of alveolar bone width and often height and bone has never been there, since alveolar bone is unlike the basal jaw bone, a development of the erupting tooth. In addition, bone quality can be more cortical and brittle than in conventional implant sites, which can influence implant placement as well as orthodontic tooth movement. The same applies to the fixed masticatory gingiva in a site with tooth aplasia, which can be narrow or missing at all. Due to these common general properties of an implant site with tooth agenesis irrespective of the number of missing teeth, this systematic review includes articles with single or few missing teeth (mild hypodontia), multiple (>6) missing teeth (oligodontia), and also total absence of teeth (anodontia).

There may be mainly four or five treatment options for occlusal rehabilitation in cases with congenitally missing teeth, including dental implant-borne prosthetics. First option is the preservation of a primary deciduous tooth. Such decision has to be made first, if a primary tooth is still present in the site due to missing eruption of a permanent successor. A second option may be the autotransplantation of other teeth, if such transplants are available. This is a well-accepted method in childhood, since tooth autotransplants can heal with a functional periodontium which enables orthodontic movement and enables the tooth to participate in growth of the alveolar crest. Furthermore, tooth transplants in childhood have a better prognosis, when root development is still incomplete and the apical foramen is still open, compared to mature teeth in adults with closed foramen. A third option is conventional prosthetics on teeth, which in childhood will typically include unsplinted overdentures, which should not interfere with jaw growth or resin-bonded bridges, since juvenile teeth should not be ground for crowns. The fourth option is dental implants. Each of the four options has its advantages and limitations and a differential indication has to be made in every single case of congenitally missing teeth. The fifth option may be orthodontic space closure as a single treatment. Of course, adjunctive orthodontic treatment is a very important part of occlusal rehabilitation in patients with congenitally missing teeth. However, this is not in the focus of this systematic review, because this therapy is not generally applicable to cases with multiple missing teeth.

To the knowledge of the authors, narrative literature reviews [4–7] and consensus meetings [8–10] have been published on the topic in the literature. There is only one systematic review without numerical meta-analysis [11]. In the latter, Yap and Klineberg addressed studies on dental implants in patients with ectodermal dysplasia (ED) and tooth agenesis but not on the alternative treatments. They found documented implant survival rates of 88.5–97.6 % in ED cases and 90–100 % in tooth agenesis. The authors concluded that implants placed in adolescent patients with ED do not have a significant negative influence on facial growth and that implants in ED patients younger than 18 years have a high risk of failure.

The aim of this study is a systematic review of the literature of treatment of patients with congenitally missing teeth and a meta-analysis in from of weighted means of survival and success data. The aim is to elucidate the role of dental implants in the group of patients with congenitally missing teeth in comparison with the other treatment options. In addition to the general treatment outcome parameters, the aim was also to include patient-centered and patient-reported outcome parameters. This paper was prepared as the basis of a consensus meeting of the German Implant Association to be held on 9th–10th of September 2015 in Aerzen, Germany.

## Methods

This systematic review was structured and performed according to the preferred reporting items of the PRISMA statement [12].

### Focused question

The focused question serving for literature search was structured according to the PICO format (Table 1) “In patients with congenitally missing teeth, does an early occlusal rehabilitation with dental implants in comparison to tooth autotransplants, conventional prosthetics on teeth or preservation of deciduous teeth have better general outcomes in terms of survival, success and better patient centered outcomes in terms of quality of life, self-esteem, satisfaction, chewing function?”.

**Table 1:** Logical deduction of the literature search phrase from the PICO questionable

Patients	Intervention	Control	Outcomes
Patients with congenitally missing teeth	Rehabilitation	Tooth autotransplants	General:
	Dental implants	Preservation of deciduous teeth	Implant/tooth survival/success
	Bone augmentation	Conventional prosthodontic treatment	Prosthesis survival/success
		Orthodontic treatment	Craniofacial growth
			Patient reported:
			Quality of life
			Self-esteem
			Satisfaction
			Chewing function
Synonyms, search terms, search phrase			
• Anodontia (MeSH)	• Rehabilitation	• Autotransplan*	• Survival
• Hypodontia	• Dental implan* (MeSH)	• Crown (MeSH)	• Success
• Oligodontia	• Alveolar bone grafting (MeSH)	• Dentur* (MeSH)	• Growth
• Tooth aplasia	• Bone augmentation	• Dental Prosthe* (MeSH)	• Quality of life (MeSH)
• Tooth agenesis		• Orthodonti* (MeSH)	• Satisfaction
• Congenitally missing teeth		• Tooth deciduous (MeSH)	• Self-esteem
• Developmentally absent teeth			• Chewing
• Birth defects			• Masticat* (MeSH)
• Noncarious defects			• Masticat* (MeSH)
• Ectodermal dysplasia			
AND (tooth OR teeth OR dental)			

Boolean operators: (Within column OR, columns AND except intervention and control OR) AND (tooth OR teeth OR dental)

NOT cancer, ophthalm*, brain, cataract

Filter: humans

Search phrase

(tooth OR teeth OR dental) AND ((anodontia OR aplas* OR agenesis OR oligodontia OR hypodontia OR developmentally absent OR congenitally missing OR noncarious OR birth defect OR ectodermal dysplasia) AND ((rehabilitation OR dental implant* OR bone augment* OR alveolar bone grafting) OR (autotransplan* OR crown OR denture* OR dental prosth* OR orthodont* OR deciduous )) AND (survival OR success OR growth OR quality of life OR satisfaction OR self-esteem OR chewing OR masticat*) NOT cancer NOT ophthalm* NOT brain NOT cataract))

### Search strategy

PubMed of the US National Library of Medicine and EMBASE were used as electronic databases to perform a systematic search for relevant articles published in the dental literature between 1980 up to 31 May 2015.

A first probatory screening using only the MeSH terms “anodontia” and “dental implants” yielded too few results. It became clear that there are numerous synonyms of anodontia, which had to be included in the search (Table 1). A search strategy based on the elements of the PICO question was constructed: (tooth OR teeth OR dental) AND ((anodontia OR aplas* OR agenesis OR oligodontia OR hypodontia OR developmentally absent OR congenitally missing OR noncarious OR birth defect OR ectodermal dysplasia) AND ((rehabilitation OR dental implant* OR bone augment* OR alveolar bone grafting) OR (autotransplan* OR crown OR denture* OR dental prosth* OR orthodont* OR deciduous )) AND (survival OR success OR growth OR quality of life OR satisfaction OR self-esteem OR chewing OR masticat*) NOT cancer NOT ophthalm* NOT brain NOT cataract)).

Screening was performed independently by the two authors. Disagreement regarding inclusion during the first and second stage of study selection was resolved by discussion.

Electronic search was complemented by an iterative hand-search in the reference lists of the already identified articles. If required, the corresponding authors were contacted and requested to provide missing data or information by email.

### Study inclusion and exclusion criteria

During the first stage of study selection, the titles and abstracts were screened and evaluated according to the following inclusion criteria:

1. English language.
2. Retrospective and prospective clinical trials, observational studies, cross sectional studies, cohort studies, case series.

During this procedure, the pre-selected publications were evaluated according to the following exclusion criteria:

1. Inclusion of minimum 5 patients (exclusion of case reports).
2. Inadequate case definition or missing follow-up times.
3. Double publication of the same sample
4. Lack of clinical data
5. Studies in cleft lip and palate patients

### Quality and risk of bias assessment of selected studies

A quality assessment of all selected full-text articles was performed. It made no sense to use the Cochrane collaborations’ tool for assessing risk of bias for randomized controlled studies since the majority of the included studies were retrospective case series. Instead, a system modified from the US Agency for Healthcare Research and Quality Methods Guide for Comparative Effectiveness Reviews was used, which asked for the sources of possible bias [13]. The criteria were each judged with low, medium, and high risk of bias: case selection bias and confounding, attrition bias (loss of participants), detection bias (reliable measures?), reporting bias (selective or incomplete reporting), followed by a summary of the risk. Exclusion and quality assessment was performed independently by both authors. Disagreements were resolved by discussion.

### Data extraction

A data extraction template was generated and based on the treatment types for the general outcome parameters and for the patient-centered outcome parameters. Due to incomplete reporting, old studies, and changing definitions in some papers, the required data on survival and success were often not found directly listed in the publications. In this case, they had to be retrieved from side informations or recalculated from tables. The following rules were applied: survival meant that the unit (implant, tooth, prosthesis) was reported to be present in the oral cavity. Success definition of an implant followed the criteria of Buser and coworkers [14] which are the following: absence of persistent subjective complaints such as pain, foreign body sensation and/or dysesthesia, absence of a peri-implant infection with suppuration, absence of mobility, absence of a continuous radiolucency (severe bone resorption) around the implant. A prosthesis either conventional or implant borne was counted as a success, if there were no complications reported like fracture, soft tissue recessions, or documented treatment needs. A deciduous tooth was counted as a success, if there was no ankylosis and infraocclusion reported. A tooth autotransplant was regarded as a success if there were no reports of ankylosis or severe root resorption, infection, or mobility.

### Statistics and data synthesis

For data synthesis, the survival and success data of the individual studies were pooled by the weighted mean method and 95 % confidence intervals were calculated as estimations of variance. A meta-analysis was not possible due to the structure of the underlying survival success data as simple percentages without a measure of variance and without control groups in most studies. The survival/success data were weighted both based on patients and units (either implants/teeth/prostheses). Because the studies showed large differences in follow-up times, these were standardized by calculating annual failure rates by dividing the success and survival data through the follow-up time in years. All spreadsheet calculations and statistics were made with the Microsoft Excel program.

## Results

### Study selection

**Fig. 1:**
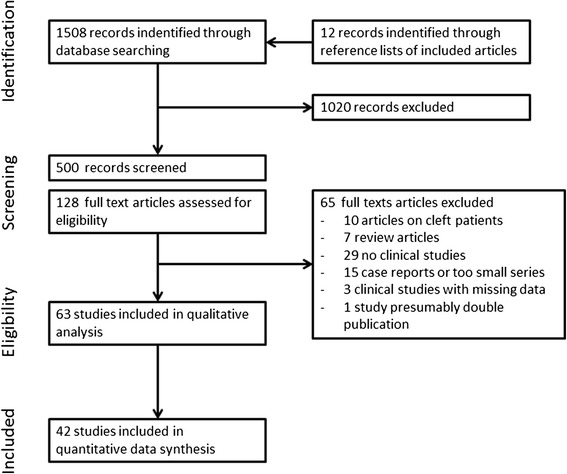
Flow diagram of literature search and inclusion

A total of 1508 potentially relevant titles and abstracts were found by the electronic search. Twelve titles found additionally by evaluation of reference lists of included articles were added. During first screening, 1020 publications were excluded based on database information. One hundred twenty-eight full-text articles were thoroughly evaluated. A total of 65 papers had to be excluded at this stage because they did not fulfill the inclusion criteria of the present systematic review. Sixty-three articles went into qualitative assessment (Fig. 1). One article had to be excluded or pooled with another because of possible double publication of the same cohort (Grecchi (b) [15]), one article because of missing follow-up times (Hvaring [16]), one because of missing clinical data (Kjaer [17]), and two articles because of missing numerical data (Dellavia [18, 19]). The study of Bergendal [20] could be kept in the analysis after contacting the author for clarification (no follow-up time reported). The implants in that study were observed only over the healing period (estimated 6 months in average). Because of too few studies and incompletely reported data studies on orthodontic gap closure, facial growth and masticatory performance were not included into the quantitative data synthesis. Forty-two studies were included in the assessment of general outcome parameter survival and success. For 16 studies on patient-centered variables, also weighted means were calculated.

### Evaluation of study quality and risk of bias

The majority (*n* = 25) of the 42 studies were retrospective, 14 were prospective including one RCT 2 were cross sectional studies, and 1 remained unclear. Despite a low evidence level in terms of study design, there were no major concerns about risk of bias. The 30/42 studies were rated with a low risk of bias, and 12/42 had a medium risk. No study had a high risk of bias and consequently no further study was excluded at this stage because of bias (Table 2).

**Table 2:** Summary of the selected studies and quality assessement

Author	Year	Study type	Case selection bias (homogeneity and confounders)	Performance bias (fidelity to protocol)	Attrition bias (loss of participants)	Detection bias (reliable measures)	Reporting bias (selective reporting or conflicting interests)	Summary assessments
								Risk of bias
Implant studies								
Ledermann [40]	1993	Retrospect	M	M	L	M	H	M
Kearns [41]	1999	ProspObs	L	L	L	L	M	L
Thilander [3]	2001	ProspObs	L	L	L	L	L	L
Guckes [42]	2002	ProspObs	M	L	L	L	M	L
Sweeney [43]	2005	Retrospect	L	L	L	L	M	L
Finnema [27]	2005	Retrospect	L	L	M	L	M	L
Poggio [44]	2005	Retrospect	M	M	M	M	M	M
Zarone [45]	2006	ProspObs	L	L	L	L	L	L
Becelli [46]	2007	Retrospect	L	L	L	L	M	L
Bergendal [20]	2008	Survey	M	L	H	M	M	M
Dueled [26]	2008	Retrospect	M	L	M	L	L	L
Krieger [25]	2009	Retrospect	M	L	L	M	M	L
Degidi [47]	2009	RCT	L	L	L	L	L	L
Creton [1]	2010	Retrospect	L	L	M	L	M	L
Grecchi [15](a), [22](b)	2010	Retrospect	H	M	M	L	L	M
Nissan [48]	2011	Unclear	H	L	M	M	M	M
Heuberer [38]	2012	Retrospect	L	L	L	L	M	L
Hosseini [49]	2013	ProspObs	L	L	L	L	L	L
Zou [50]	2014	Retrospect	M	L	L	L	L	L
Autotransplants								14/19 L 5/19 M
Kristersson [51]	1991	Retrospect	M	L	L	L	L	L
Kugelberg [52]	1994	ProspObs	M	L	L	L	L	L
Marcusson [53]	1996	ProspObs	M	L	L	L	L	L
Josefsson [54]	1999	Retrospect	M	L	M	L	L	L
Czochrowska [55]	2002	Retrospect	H	L	M	L	L	M
Bauss [23]	2004	prospCT	M	L	M	M	M	M
Jonsson [56]	2004	ProspObs	M	L	L	L	M	L
Tanaka [57]	2007	Retrospect	M	L	M	L	M	M
Mensink [58]	2010	Retrospect	M	L	L	L	M	L
Kvint [59]	2010	Retrospect	M	L	L	L	L	L
Bokelund [24]	2013	Retrospect	L	L	L	L	L	L
Deciduous teeth								8/11 L 3/11 M
Bjerklin [60]	2000	ProspObs	M	L	M	L	L	L
Ith-Hansen [61]	2000	ProspObs	L	L	L	L	L	L
Sletten [62]	2003	Retrospect	L	L	M	L	M	L
Bjerklin [63]	2008	ProspObs	M	L	M	L	L	L
Kjaer [17]	2008	Retrospect	L	L	M	L	L	L
Hvaring [16]	2013	Cross section	L	L	L	L	L	L
Convent. Prosth.								2/6 L 4/6 M
Hobkirk [64]	1989	Retrospect	H	M	M	L	M	M
Pröbster [65]	1997	Retrospect	M	L	M	L	M	M
Garnett [66]	2005	Retrospect	M	L	M	L	M	M
Dueled [26]	2008	Retrospect	M	L	M	L	L	L
Krieger [25]	2009	Retrospect	M	L	L	M	M	M
Spinas [67]	2013	ProspObs	L	L	L	L	M	L
								2/6 L 4/6 M
Total	*n* = 42	25 retrosp 14 prosp						30/42 L12/42 M

### Studies on general outcome parameters

#### Studies on dental implants and implant-supported prosthetics

A total of 19 studies with a mean follow-up time of 4.6 years (maximum 15.1 years) was included (Table 3). Most studies were of retrospective character. The heterogeneity of the studies concerning survival and success data was low and acceptable for the numerical data synthesis. The studies showed some heterogeneity in patient inclusion, because in some studies, missing single teeth and treatments with single crowns on implants were mixed with severe oligodontia and varying prosthetic treatment including overdentures on implants. Also, management of bone defects and necessity of bone grafting were heterogenous between the studies. This heterogeneity did not lead to exclusion from the present numerical evaluation. The study of Bergendal and coworkers [20] was considered important, because a high ratio of implant failures was reported in childhood. It was one of the very few studies in the literature on implantation in childhood. However, no follow-up time was reported because it was a cross-sectional survey. The author was contacted via email and confirmed that all implant losses had occurred during healing time before prosthetic restoration. Since healing time was has usually a maximum of 6 months, the follow-up time was set on arbitrary 0.6 year. The study of Durstberger et al. [21] had to be excluded because of vague follow-up data and no information of implant losses during follow-up and most of the mentioned patients were only planned for implant placement but had no implants placed. The studies of Grecchi (a) [22] were pooled with Grecchi (b) [15] for numerical analysis, because of assumed double publication of the same samples. In most of the studies, implants had been placed in conjunction with bone grafting, but only Grecchi (a) [22] had presented outcome data on this aspect. There were no prominent differences for implants placed in grafted bone compared with non-grafted sites. Secondary infraocclusion of approximately 1 mm was a problem of implant placements in the maxillary incisor region in childhood [3]. Marginal bone resorption at implants was not a prominent finding and ranged from 0.2 to 1.2 mm in the studies which reported this aspect. The success/survival data and further subgroup analysis for implant survival data is presented below.

**Table 3:** Synopsis of included studies on dental implants and prosthetics on dental implants in order of publication year

Author		Year	Study type	Population	Treatment	Comparison	Patients	Implants	Implant survival [%]	Implant success [%]	Prosthes. survival [%]	Marginal resorpt.[mm]	Infra occlusion [mm]	Follow-up [y]	Risk of bias
Ledermann [40]	1993		Retrospect	Agenesis, trauma	FPD	9–18 years	34	42	90	90	100			3	M
Kearns [41]	1999		ProspObs	ED 5–17 years	Full denture	All	6	41	97.6					7.8	L
						<6 years	2	9	100	44.4	0			7.8	
						>12 years	4	32	96.9	96.9	100			7.8	
Thilander [3]	2001		ProspObs	Agen,<18 years trauma	SC, FPD	All	18	47	100	100	93.9			10	L
						Incisors	12	26	100	100	88.2	0.75	0.98	10	
						Canines	4	8	100	100	100	0.6	0	10	
						Premolars	5	13	100	100	100	0.5	0.1–0.6	10	
Guckes [42]	2002		ProspObs	ED 5–18 years	Full denture	All	51	264	90					2	L
						Maxilla		21	71					2	
						Mandible		243	91					2	
						<11 years		46	85					2	
						11–18 years		122	87					2	
						>18 years		96	95					2	
Sweeney [43]	2005		Retrospect	ED adolesc.	FPD, full denture	All	14	61	88.5		94			3.33	L
						Maxilla	4	15	80					3.33	
						Mandible	14	46	91.3					3.33	
Finnema [27]	2005		Retrospect	Oligodont. adults	FPD	All	13	87	89.7					3	L
						Maxilla			86					3	
						Mandible			96					3	
Poggio [44]	2005		Retrospect	Oligodont. adults	SC	No	24	46	100		82.6			5.25	M
Zarone [45]	2006		ProspObs	Ag incisors adults	SC	No	30	34	97.1	94.1		1.2		3.75	L
Becelli [46]	2007		Retrospect	Oligodont. adults	SC, FPD	No	8	60	96.6					8.5	L
Bergendal [20]	2008		Survey	ED, adoles children	n.s.	All	26	47	76.6					0.6	M
						14–15 years	21	33	93.9					0.6	
						<13 years	5	14	35.7					0.6	
Dueled [26]	2008		Retrospect	Oligodont	SC, FPD	Implant supported	110	179	100	87.3	99			3.8	L
Krieger [25]	2009		Retrospect	Oligodont. adults	SC, FPD	All impl	17	40	92.5		87.9			15.1	L
						SCImpl	12	24	87.5	62	83.3			15.1	
						FDP Impl	6	16	100	100	100			15.1	
Degidi [47]	2009		RCT	Ag Incisors adults	SC	All	60	60	100	98.3	100			3	L
						Imm. load	30	30	100	100	100	0.85		3	
						Conv. load	30	30	100	96.6	93.3	0.75		3	
Creton [1]	2010		Retrospect	Oligodont. adults	FPD	No	44	214	89.8					2.9	L
Grecchi (a) [15]	2010		Retrospect	ED adults	FPD	All	4	44	100	90.9		0.4		1.8	M
						Grafted	3		100			0.3			
						Non-graft	1		100			1.2			
						Maxilla		20	100			0.2			
						Mandible		24	100			0.7			
Grecchi (b) [22]	2010		Retrospect	ED adults	FPD	No	8	78	98.7			0.5–0.6		1.8	Pooled
Nissan [48]	2011		Unclear	Agenesis	SC	No	12	21	95.2	95.2				2.5	M
Heuberer [38]	2012		Retrospect	Mixed ED < 12 years	Full denture	All	6	16	93.8		100			4	L
						Maxilla, onplants	4	8	87.5		100			5	
						Mandible	3	8	100		100			3	
Hosseini [49]	2013		ProspObs	Hypodontia	SC	No	59	98	100	99	97.2			3	L
Zou [50]	2014		Retrospect	ED Adults	FPD	No	25	169	98.3	97.2	100	1.4		5	L

#### Studies on tooth autotransplantation

A total of 11 studies with a mean follow-up time of 7.6 years (maximum 26.4 years) were included, all of which qualified also for the numerical analysis (Table 4). The study heterogeneity concerning survival and success data was low and acceptable for numerical data analysis. The studies showed some heterogeneity in patient inclusion, because in most studies a tooth agenesis sample was mixed also with missing teeth due to trauma and other reasons, which did not lead to exclusion from the present evaluation. According to the table, three studies found a better survival between 10 and 46 % of immature teeth compared to mature teeth. The Bauss study [23] demonstrated better pulpal and periodontal conditions if a transplantated tooth was not moved orthodontically, in comparison of the different orthodontic treatments derotation was more harmful than extrusion. According to the Bokelund study [24], premolar transplants performed better than molars. A preceding primary tooth at the receptor site of a tooth autotransplant was connected with a lower transplant success than a normal primary tooth. The numerical success/survival data are presented below.

**Table 4:** Synopsis of included studies on tooth autotransplantation in order of publication year

Author	Year	Study type	Population	Comparison	Patients	Transplants	Transplant survival [%]	Transplant success [%]	Pulp vitality [%]	Intact periodont. [%]	Follow-up [y]	Risk of bias
Kristersson [51]	1991	Retrosp.	Agen, incis, trauma	All	50	50	98	82			7.5	L
				Immature		41		90			7.5	
				Mature		9		44			7.5	
Kugelberg [52]	1994	Prosp.Ob	Ag, Tr	All	40	45	96	89			4	L
				Immature		23		96	100	96	4	
				Mature		22		82	0	82	4	
Marcusson [53]	1996	Prosp.Ob	Ag, missing	No	29	31	87				8	L
Josefsson [54]	1999	Retrosp.	Oligodontia	All	80	110	91				4	L
				Immature		11		92			4	
				Mature		99		82			4	
Czochrowska [55]	2002	Retrosp.	Agenesis	No	28	33	90	79			26.4	M
Bauss [23]	2004	Retrosp.	Ag, missing	All	88	91	100	84.6			4	M
				OrthDerot	27	28	100		71.4	67.8	4	
				OrthExtru	20	21	100		90.5	85.7	4	
				NoOrthod	41	42	100		97.6	95.2	4	
Jonsson [56]	2004	Prosp.Ob	Ag, missing	No	32	40	92.5		76	100	10	L
Tanaka [57]	2007	Retrosp.	Ag, missing	No	24	28	100		60.7		4	M
Mensink [58]	2010	Retrosp.	Ag, missing	No	44	63	100		89		1	L
Kvint [59]	2010	Retrosp.	Ag, missing	No	215	215	88.4	81			4.8	L
Bokelund [24]	2013	Retrosp.	Agenesis	All	157	211	100				10	L
				Premolar		162		93		93	10	
				Molars		49		60		60	10	
				NormalPrim		71		95		95	10	
				InfraoccPrim		140		87		87	10	

#### Studies on preservation of deciduous teeth

A total of 6 studies with a mean follow-up time of 12.5 years (maximum 16.5 years) was included, of which only 4 qualified for the numerical analysis (Table 5). The studies were homogenous in terms of outcome parameters and inclusion of only tooth agenesis patients. Two studies (Kjaer [17], Hvaring [16] ) contained no data on follow-up times. The Kjaer study made an important statement that in patients in whom the dentitions shows morphological signs of ectodermal dysplasia (screw driver shaper incisors, taurodontism, invaginations of incisors or slim incisors, typical ED sites of aplasia), the risk of root resorption fatal prognosis of a deciduous tooth was elevated by the factor 1.46 compared to patients with normal tooth appearance. In general, root resorption, ankylosis, and consecutive infraocclusion were a problem of preservation of deciduous second molars at the site of a secondary premolar aplasia. The absence of root resorption was interpreted as success criterion in most studies, therefore, in contrast to high survival figures, the success of deciduous teeth was lower. The numerical success/survival data are presented below.

**Table 5:** Synopsis of included studies on preservation of deciduous teeth in order of publication year

Author	Year	Study type	Population	Comparison	Patients	Deciduous teeth	Deciduous tooth survival [%]	No Infra-occlusion (success) [%]	No root resorption [%]	Odds ratio	Follow-up [y]	Risk of bias
Bjerklin [60]	2000	ProspObs	Agenes	no	41	59	88	45	40		9	L
Ith-Hansen [61]	2000	ProspObs	Agenes	no	18	26	89.6	88.4	88.4		16.5	L
Sletten [62]	2003	Retrospect	Agenes	No	20	28	86				12.4	L
Bjerklin [63]	2008	ProspObs	Agenes	No	99	149	91	48	16.7		12.2	L
Kjaer [17]	2008	Retrospect	Agenes	ED shaped versus normal	105	n.s.				1.46	No	L
Hvaring [16]	2013	Cross section	Agenes	No	111	188		57.4	66.6		No	L

#### Studies on conventional prosthetics on teeth

A total of 6 studies with a mean follow-up time of 7.2 years (maximum 15.1 years) was included, all of which qualified for the numerical analysis (Table 6). This relatively small group of studies showed a high level of heterogeneity in patient inclusion, because this group comprised various indications from single missing incisors up to severe hypodontia treated with overdentures on teeth. Nevertheless, it was decided to keep these results in the numerical analysis, because of relatively consistent success and survival data across the different prosthetic therapies. The numerical success/survival data are presented below.

**Table 6:** Synopsis of included studies on conventional prosthetics on teeth in order of publication year

Author	Year	Study type	Population	Treatment	Comparison	Patients	Teeth	Prostheses	Prosthesis survival [%]	Prosthesis success [%]	Follow-up [y]	Risk of bias
Hobkirk [64]	1989	Retrospect	Hypodontia	Overdenture	All	138		138	51.5		6	M
					Mandible	48		48	33		6	
					Maxilla	90		90	61		6	
Pröbster [65]	1997	Retrospect	HypodTrauma	Single crown	No	264	392	325	60		10	M
Garnett [66]	2005	Retrospect	Hypodontia	Single incis crown		45	73	73	59		6	M
Dueled [26]	2008	Retrospect	Oligodont adults	SC, FPD	On teeth	19		30	89.5	80	3.8	L
				SC, FPD	On implants	110		179	99	91	3.8	
Krieger [25]	2009	Retrospect	Hypodontia	All	On teeth	5		25	85.7	42	15.1	M
				Single Cr.	On teeth	5		5	80	80	15.1	
				FDP	On teeth	10		20	84.1	72.8	15.1	
				All	On implants	17		33	87.9	53	15.1	
				SC	On implants	12		24	83.3	62.5	15.1	
				FDP	On implants	6		9	100	66.7	15.1	
Spinas [67]	2013	Prosp.Obs.	Hypodontia	Resin bridge single Incis		30	32	32	93.8		5	L

### Data synthesis

#### Comparison of the four treatments

The results of the weighted mean method and annual failure rates are presented in Table 7. In addition, the results are visualized in Figs. 2, 3, 4, 5, 6, 7, 8, and 9.

**Table 7:** Numerical results of general outcome parameters (weighted mean values)

[%]	Survival patient weighted	95 % confid. interval	Success patient weighted	95 % confid. interval	Survival impl/tooth/unit weighted	95 % confid. interval	Success impl/tooth/unit weighted	95 % confid. interval	Annual failure rate survival patient weighted	95 % confid. interval	Annual failure rate success patient weighted	95 % confid. interval	annual failure rate survival impl/tooth/unit weighted	95 % confid. interval	Annual failure rate success impl/tooth/unit weighted	95 % confid. interval
Treatment comparisons																
Implants	95.3	1.9	92.7	4.6	94.1	1.8	95.6	4.1	3.317	0.196	0.815	0.055	3.280	0.150	1.987	0.159
AutoTX	94.4	4.1	82.5	11.7	94.8	4.0	85.0	4.0	1.061	0.112	1.832	0.638	1.167	0.100	2.334	0.184
Deciduous	89.6	19.1	51.8	12.6	89.7	19.8	51.7	13.1	0.908	0.157	2.928	1.472	0.903	0.161	4.333	1.494
ConvProsth	60.2	9.4	59.4	44.3	62.6	7.6	59.4	44.3	5.144	0.851	5.368	4.947	11.098	1.477	9.863	6.895
Implant subgroup analysis																
Children	72.4	18.8	44.4	n.a.	80.1	17.6	44.4	n.a.	50.177	32.083	7.128	n.a.	25.532	9.836	7.128	n.a.
Adolescents	93.0	9.5	93.7	27.0	82.0	8.3	95.7	7.5	4.610	1.029	2.052	1.313	4.626	0.712	1.262	0.724
Adults	97.4	4.0	92.2	7.5	95.6	3.3	91.4	6.8	0.670	0.001	1.850	0.272	1.280	0.122	2.188	0.276
Ectod.Dyspl.	89.6	8.4	93.4	30.3					11.665	2.520	1.431	0.219				
Hypo/oligodo	97.2	3.9	92.4	5.4					0.864	0.067	1.899	0.199				
Prosthesis on implants	97.8	2.3	94.5	2.4					0.864	0.032	0.884	0.029				
Prosthesis on teeth	61.4	7.9	77.9	19.2	62.1	7.7	77.4	20.6	5.060	0.726	3.666	1.695	12.111	1.496	3.046	1.650
Sing. crowns	98.5	24.5														
FPD	96.3	7.7														
Full dentures	90.6	9.0														
Maxilla					84.2	8.3										
Mandible					91.9	30.3										
Single aplas	99.1	14.5														
Mild hypodo	94.6	5.3														
Severe Oligo	93.1	11.0

**Fig. 2:**
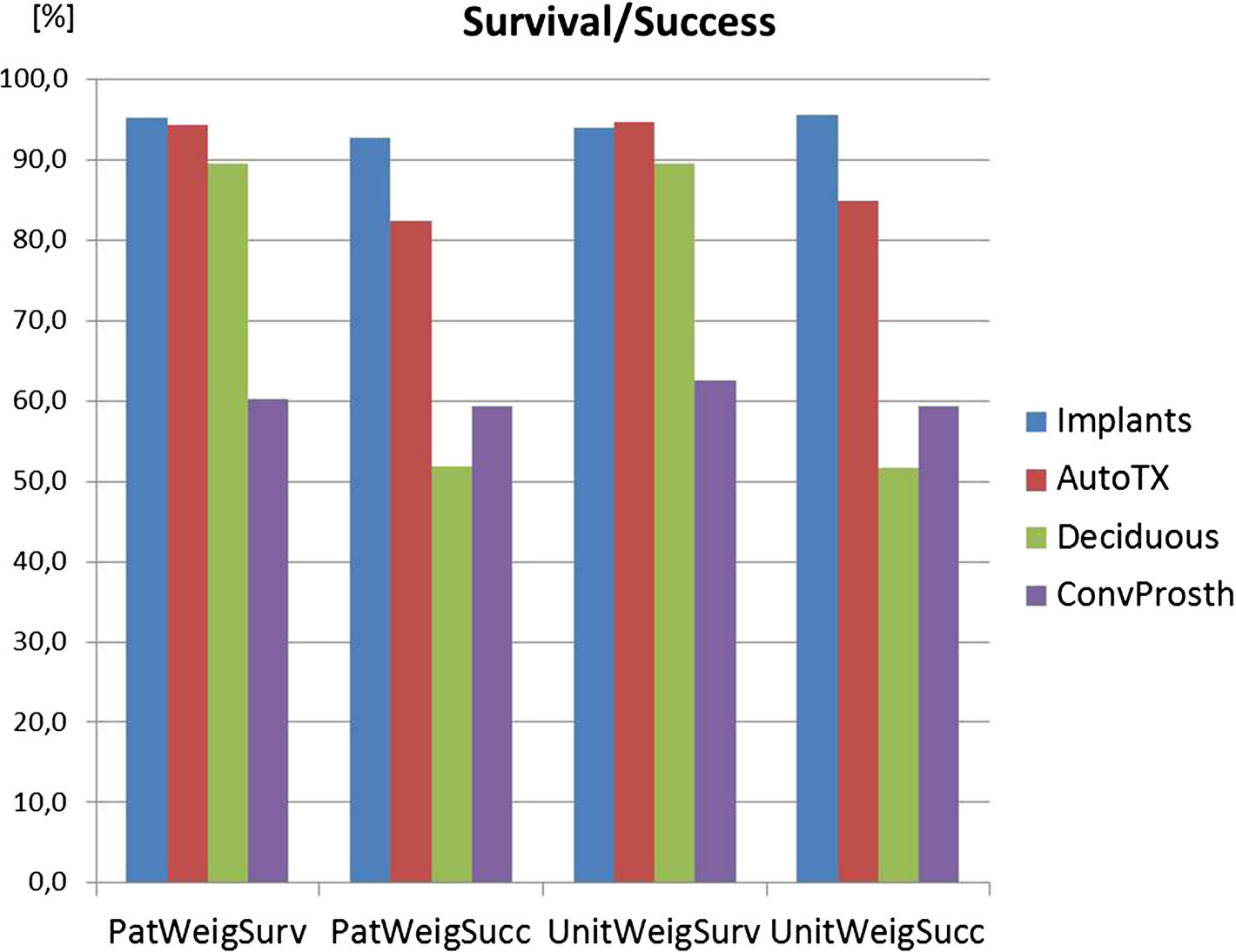
The diagram shows survival and success data of implants, tooth autotransplants, preserved deciduous teeth, and conventional prostheses on teeth. The means of the underlying studies are displayed either weighed on patients’ base (=PatWeigSurv, PatWeigSucc) or on units’ base (implant, tooth, or prosthesis) (=UnitWeigSurv, UnitWeigSucc)

**Fig. 3:**
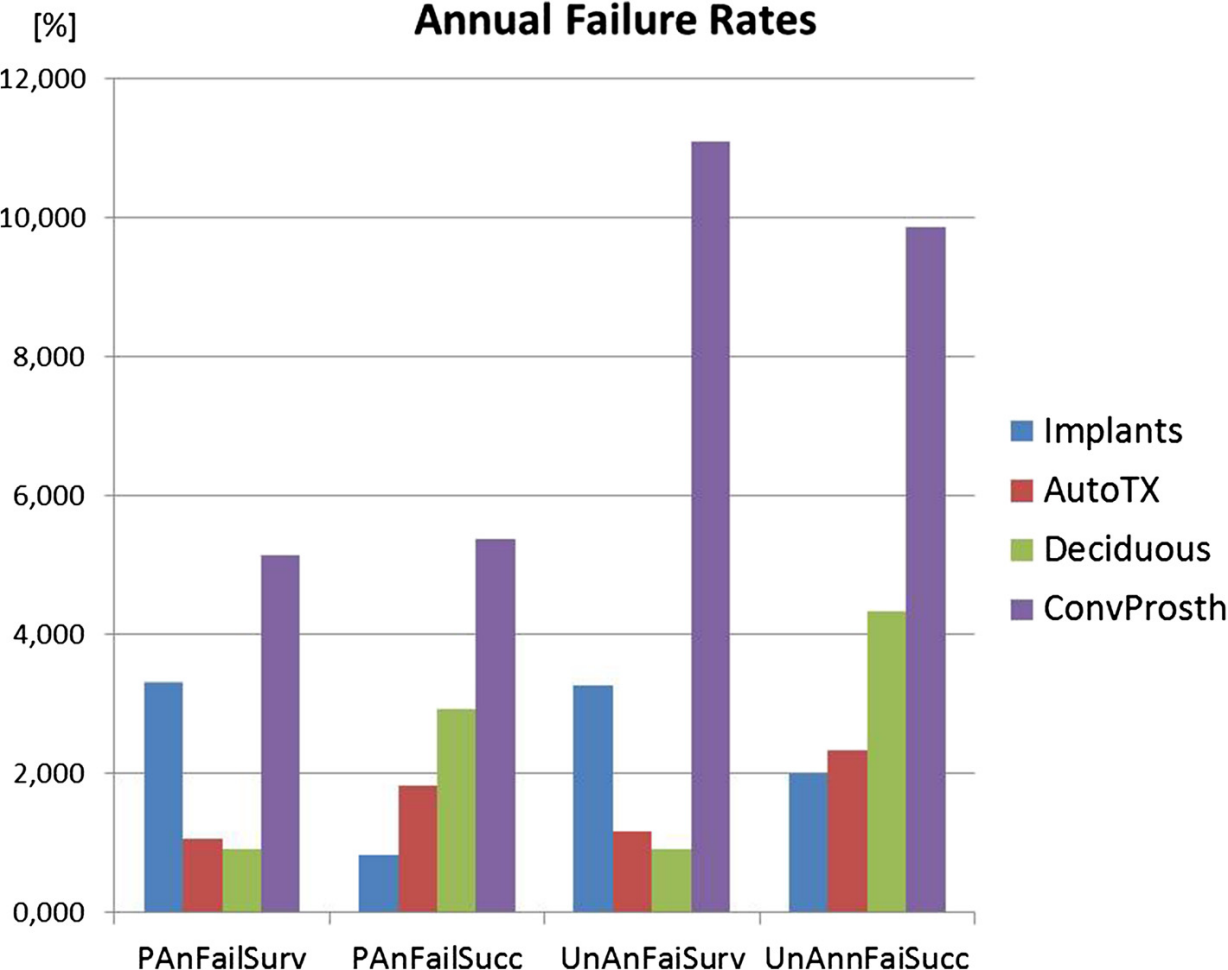
The diagram shows annual failure rates of the four treatments (implants, tooth autotransplants, preserved deciduous teeth, and conventional prostheses on teeth). The data are differentiated as in Fig. 2, either weighed on patients’ base (=PAnFailSurv, PAnFailSucc) or on units’ base (implant, tooth, or prosthesis) (=UnAnFaiSurv, UnAnnFaiSucc)

**Fig. 4:**
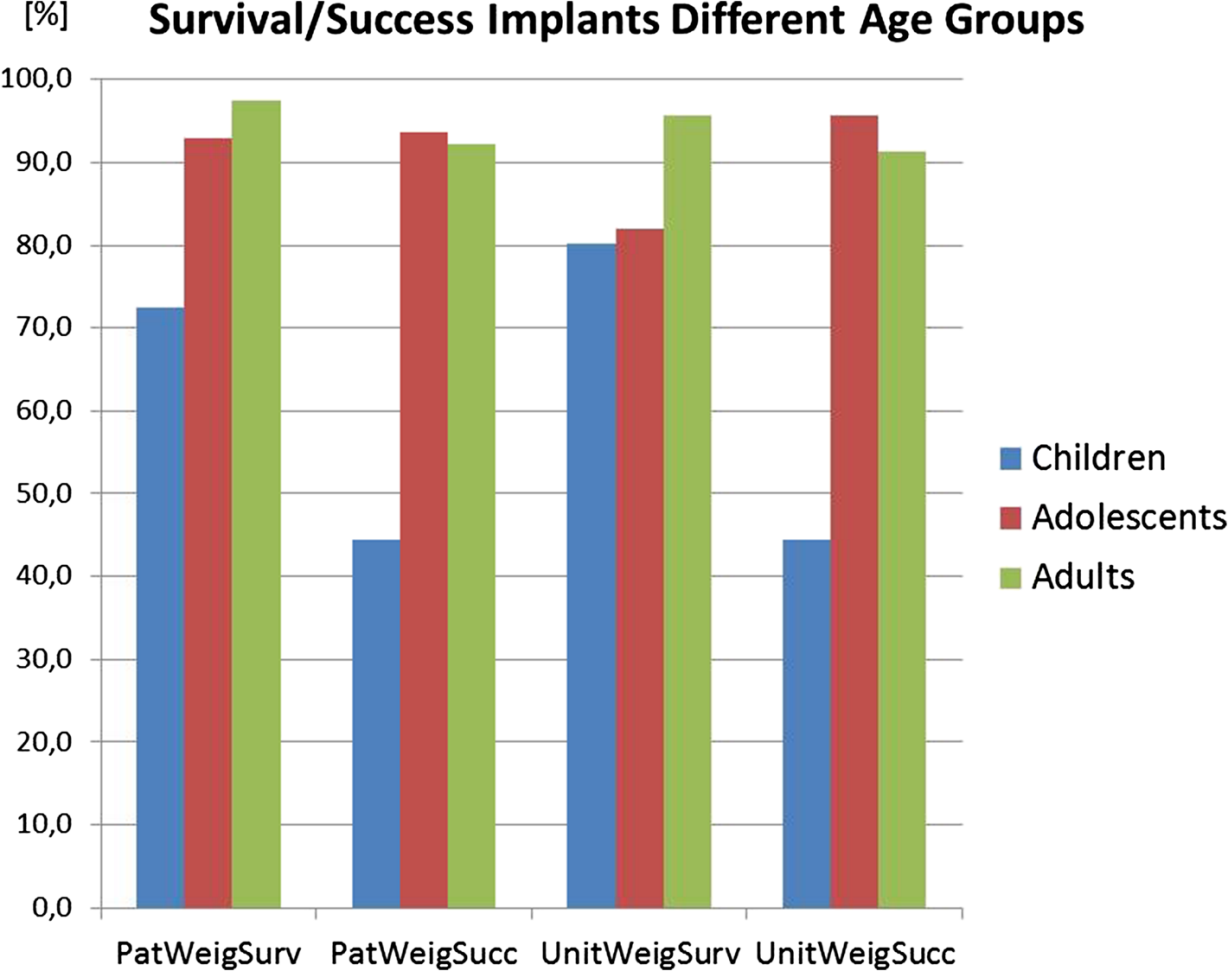
The diagram shows the same data types as Fig. 2, however here differentiated according to three age groups, children <13 years, adolescents <18 years, and adults >18 years

**Fig. 5:**
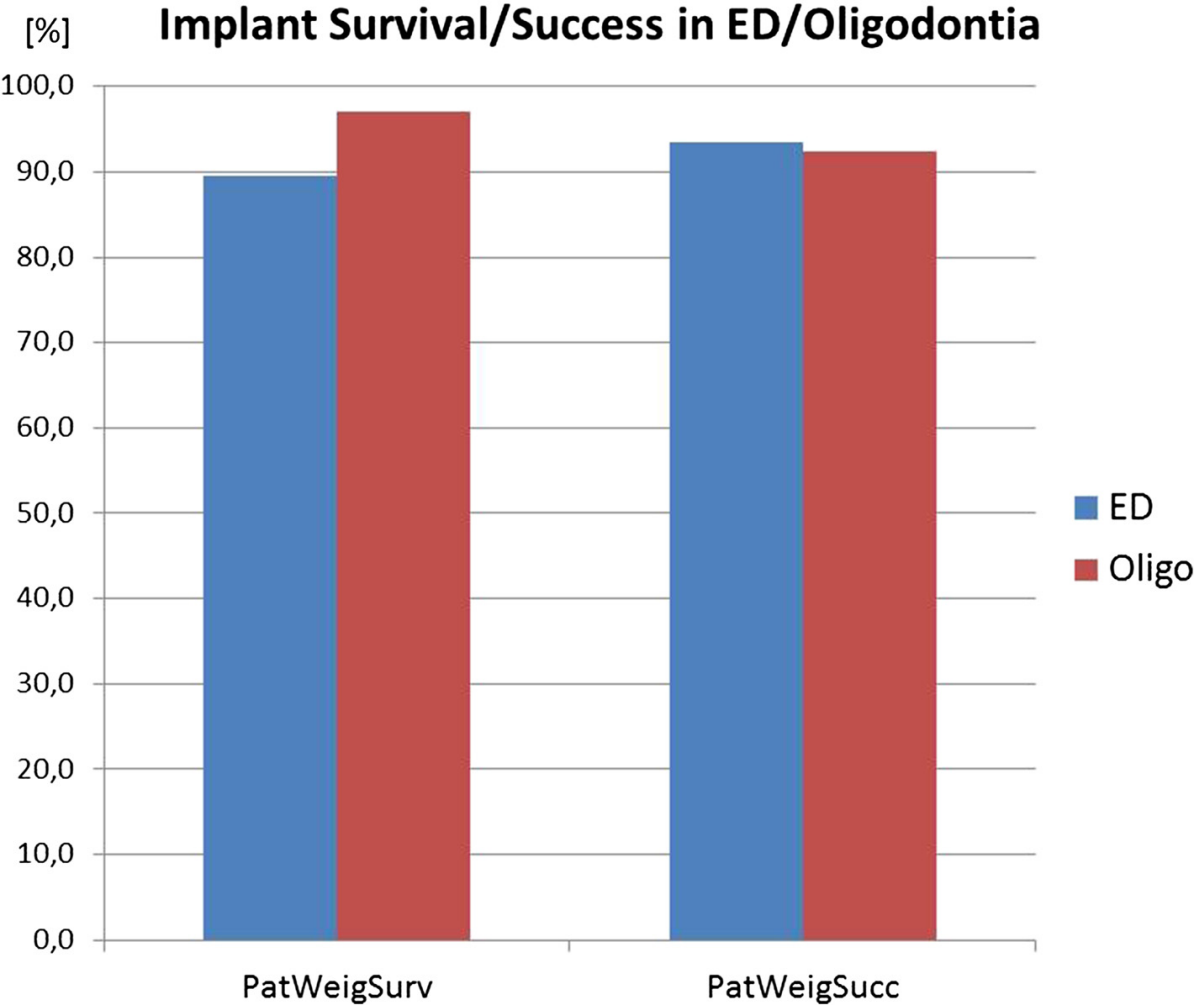
Only slight differences in implant prognosis were observed between studies with syndromic (ED) and non-syndromic cases

**Fig. 6:**
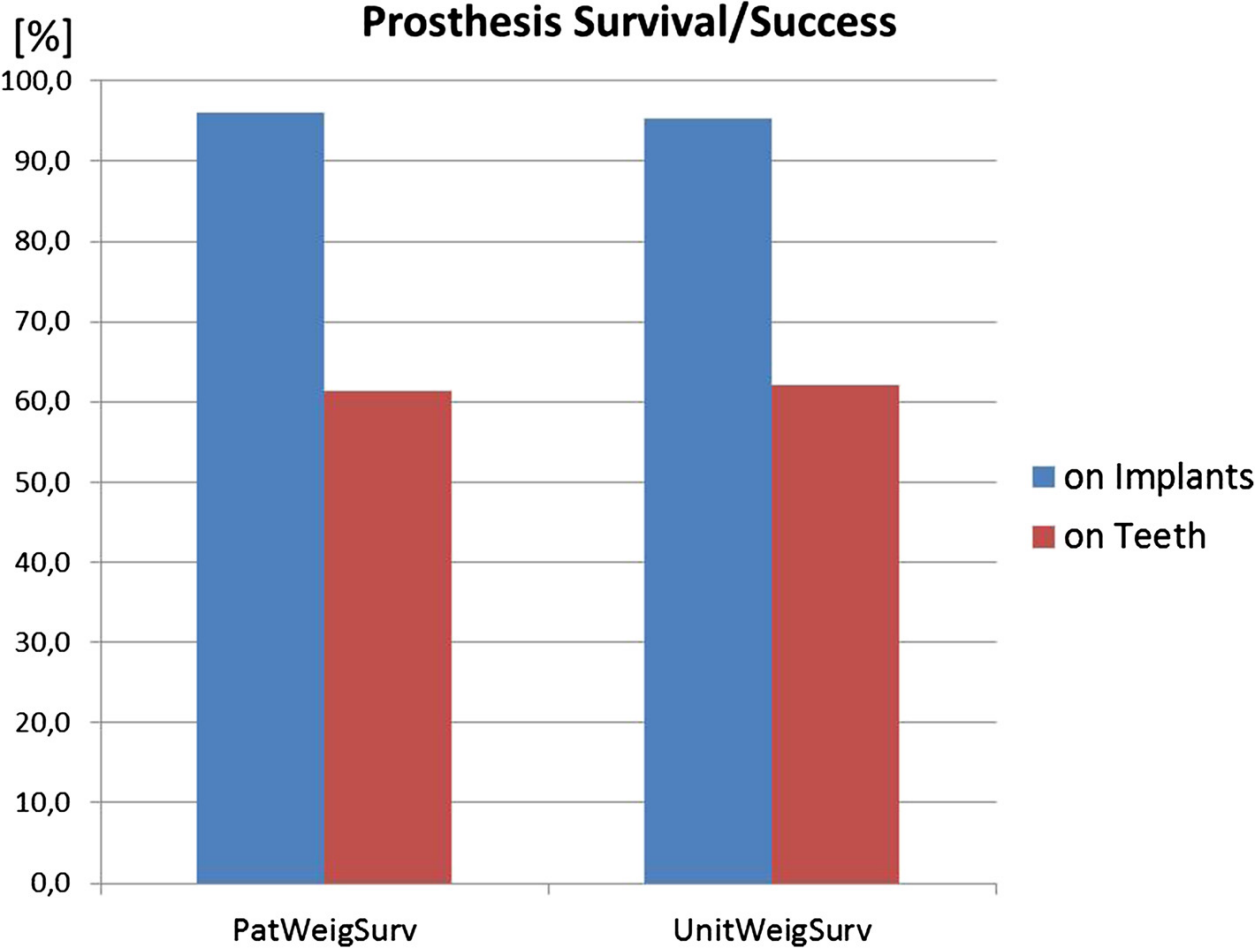
Prostheses on implants had a better prognosis than prostheses on teeth

**Fig. 7:**
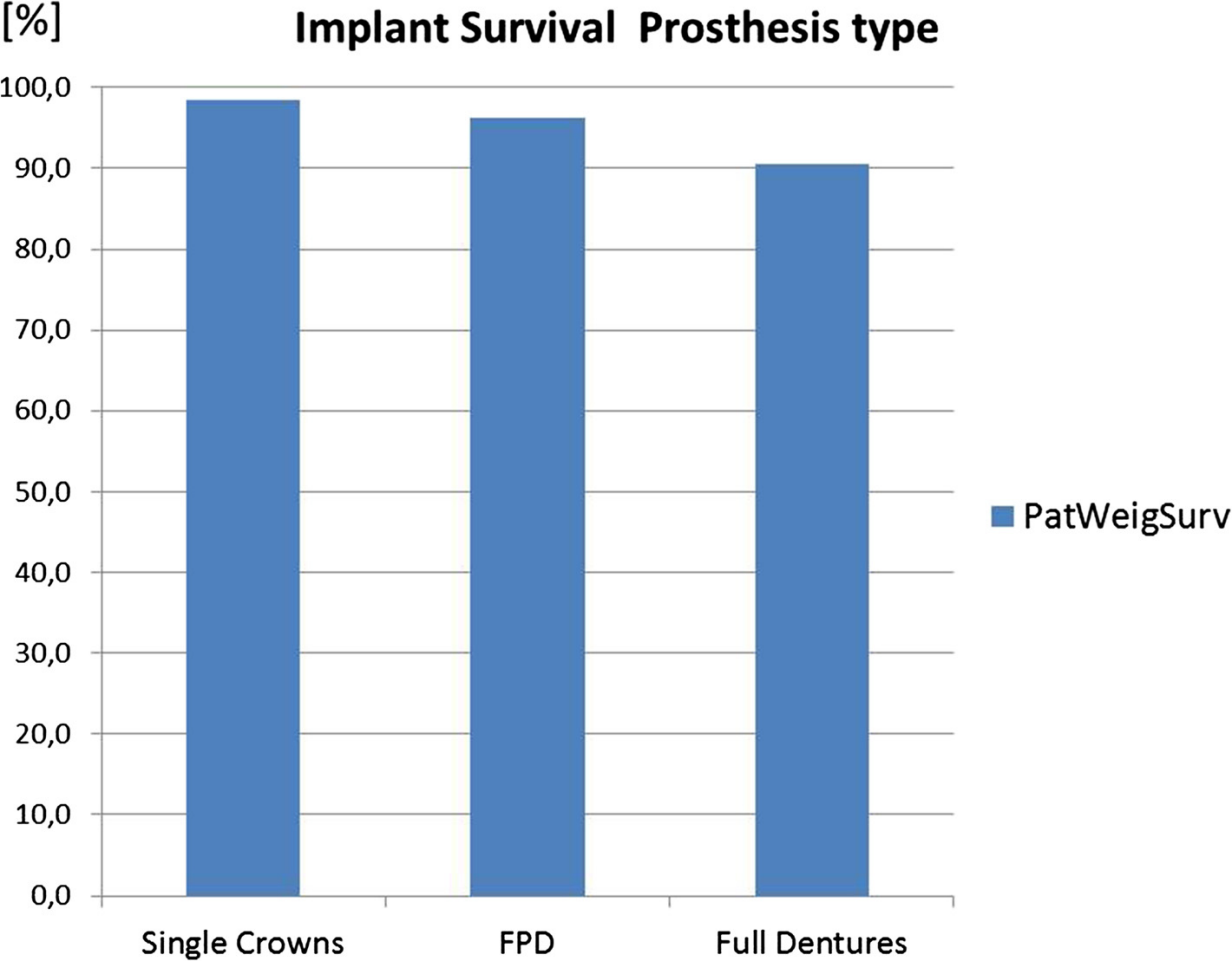
Compared to the difference between prostheses on implants and teeth (Fig. 6), the type of prosthesis plays a minor role on implant survival

**Fig. 8:**
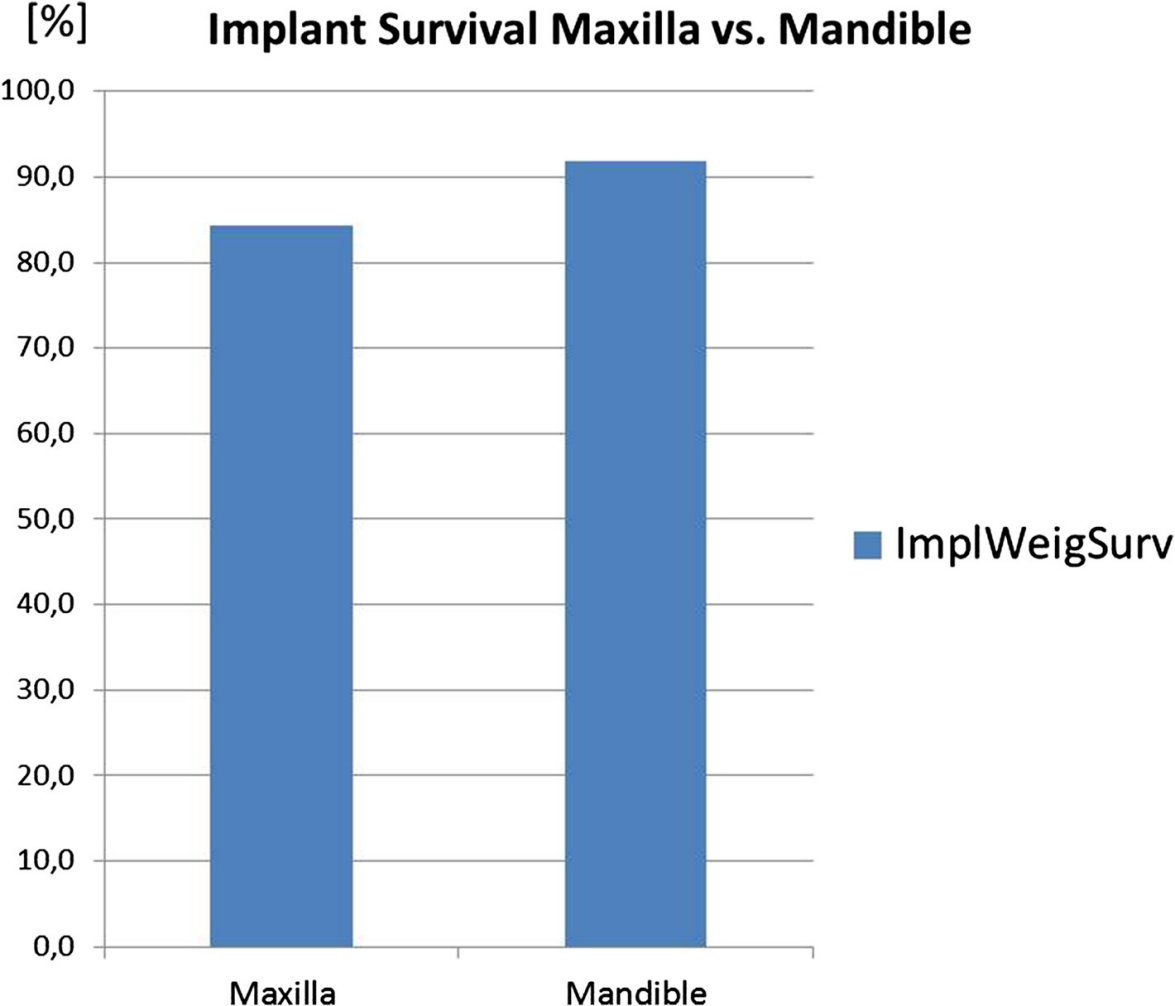
Implants in the maxilla had a lower prognosis than implants placed in the mandible

**Fig. 9:**
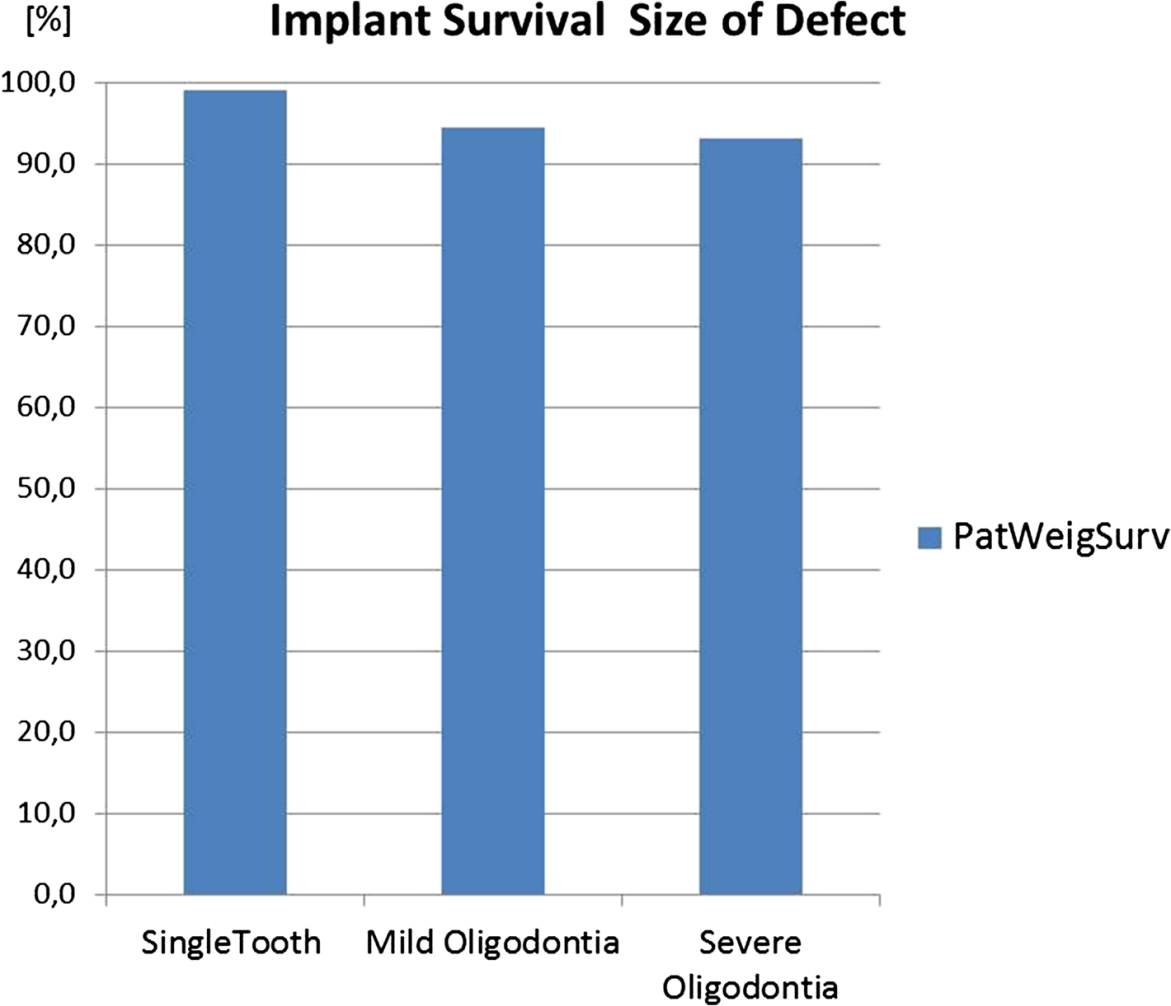
Compared to the difference between prostheses on implants and teeth (Fig. 6), the size of the defect either single missing teeth or mild or severe hypodontia plays a minor role for implant survival

There is a marked difference in survival data if implants, autotransplants, and preservation of deciduous teeth are compared with conventional prosthetics on teeth, which has only a prosthesis survival of 60.2 % (CI 9.4) at a mean follow-up of 8.4 years. If not the patient but implants/teeth or prosthetic units are used to weigh the means, the results do not change markedly (Fig. 2).

Looking upon the success data, both deciduous teeth and autotransplants have markedly lower success than survival, which is mainly due to the high rate of ankyloses and root resorption in both treatment options using the natural teeth (Fig. 2).

Implants have higher annual failure rates than the natural tooth alternatives. This is also an effect of the shorter follow-up times in implants (4.6 years) compared to autotransplants (7.6 years) and deciduous teeth (12.5 years) (Fig. 3).

A direct comparison of implant and conventional prosthetics was made in two retrospective studies of Krieger and coworkers [25] and Dueled and coworkers [26]. In both studies, prostheses on implants had better survival than on teeth. In the Dueled study, the difference of the prosthesis survival rates between implants and teeth was 9.5 % and the success rates differed by 11 % in favor of implants. The respective differences in the Krieger study were a survival difference of 2.2 % and a prosthesis success difference of 11 %. Krieger and coworkers observed also marked differences of >40 % between prosthesis success and survival, as an indicator for prosthetic maintenance and repair needs over a very long follow-up time of 15.1 years.

### Subgroup analysis of dental implant treatment

#### Children versus adolescents versus adults

There is a clearly lower implant survival with 72.4 % (CI 18.8) when dental implants are used in childhood below the age of 13 (Fig. 4). In most included studies, implant losses in children occurred early during the healing phase. Also in adolescents below the age of 18 years with a success of 93.0 % (CI 9.5) dental implants performed slightly lower than in adults with a success of 97.4 % (CI 4.0) (Fig. 3). The annual implant failure rates in children were 50.177 % (CI 32.083), in adolescents 4.610 % (CI 1.029) and in adults 0.670 % (CI 0.001) (Table 7). The mean observation time of the here included studies in children was 4.1 year, in adolescents 4.9 years, and in adults with congenitally missing teeth 6.4 years.

#### ED versus non-syndromic congenitally missing teeth (oligodontia)

The implant success/survival data of ED patients are slightly lower than of non-syndromic patients (Fig. 5). The difference is more marked looking upon annual failure rates (Table 7), indicating that these losses occurred after short observation times. These losses were observed mainly during healing time in children suffering from ED.

#### Prosthesis survival on teeth versus implants

The prosthesis survival on teeth is 61.4 % (CI 7.9) after a mean observation time of 7.2 years. On implants, this figure is markedly higher with 97.8 % (CI 2.3) prosthesis survival after a mean observation of 4.6 years (Fig. 6).

#### Prosthesis type

The survival data of implants restored with single crowns are slightly higher than restored with fixed partial dentures than restore with full dentures (Fig. 7).

#### Maxilla versus mandible

The mandible shows with 91.9 % (CI 30.3) a better implant prognosis in patients with congenitally missing teeth than the maxilla with 84.2 % (CI 8.3) (Fig. 8).

#### Size of defect

Patients with single tooth aplasias (99.1 % (CI 14.5)) had better implant survivals than patients with mild hypodontias (94.6 (CI 5.3) and patients with severe hypodontias (93.1 (CI 11.0)) (Fig. 9).

### Studies on patient-centered outcome parameters

#### Studies on quality of life, self-esteem, and patient satisfaction

A total of 16 retrieved studies included numerical assessment data of patient-centered parameters (Table 8). Many studies were cross-sectional studies with description of a baseline situation in hypodontia patiendraw 1ts. The patient inclusion was relatively homogenous between the studies. However, the studies were relatively heterogenous in the outcome parameters and study treatments because implant, conventional prosthetism, autotransplants, and orthodontic gap closure were included in this table. The number of studies in each treatment was in some cases *n* = 1 making numerical comparisons difficult.

**Table 8:** Synopsis of included studies on patient centered outcome parameters in order of publication year

Autor	Year	Study type	Population	Treatment	Comparison	Patients	Implants/missing tooth units	Baseline OHIP [scores]	OHIP improvement [scores]	OHIP esthetic [scores]	Rosenberg self-esteem [scores]	Self-esteem improvement [%]	CPQ [scores]	Satisfact [% sample or vas [%]	Risk of bias
Marcusson [53]	1996	Retrospective	Agenesis missing	AutoTX	no	29	31							75	M
Robertsson [29]	2000	Cross section	Agen lat inc	Orthod versus bridge	Orthod clos	30	30							66.5	L
					Orthod open	20	20							69.5	
Finnema [27]	2005	Retrospective	Oligodontia	FPD implants	Posttreatment	13	87					61		80	M
Wong [68]	2006	Cross section	Oligodontia	n.r.	No	25	93						29		M
Stanford [69]	2008	Cross section	ED	FPD implants	Posttreatment	109	624							91	M
Dueled [26]	2008	Retrospective	Oligodont	SC, FPD	All	129	215	13.4							M
					Control			8.2		14				96	
					Implants	110				41				98	
					Convent.	19				47				84	
Locker [70]	2010	Cross section	Oligodontia	n.r.	No	36	245						22.3		
Laing [71]	2010	Cross section	Oligodontia	n.r.	Oligodontia all	62							26.8		L
					Normal control	61							28.5		
Goshima [28]	2010	ProspObs	Hypodontia	Implant SC	No	18	37	35.2	−26.3						L
Kohli [72]	2011	Cross section	ED	n.r.	11–14 years	35	n.g.						25.1		L
					15–19 years	14							35.9		
					Self perceived								31.6		
					By caregivers								35.0		
Meaney [73]	2012	Cross section	ED	n.g.	no	10	n.g.	61							L
Hosseini [49]	2013	ProspObs	Hypodontia	Implant SC	No	59	98	16.2	−8.3						L
Hashem [37]	2013	Cross section	Oligodontia	n.g.	ED	41	n.g.	62			22				M
					Normal control	41	n.g.	31			22.3				
Anweigi (a) [74]	2013	Long Prosp Obs	Hypodontia	Bridges orthod.	FPD completed	40	n.g.	35	−19.5						L
					Orthodontic phase	37	n.g.	32	22						
Anweigi (b) [75]	2013	Cross section	NonsyndrOligodonti	n.g.	16–18 years	40	n.g.	28							L
					19–34 years	42	n.g.	33.5							
Zou [50]	2014	Retrospective	ED	Implants	No	25	169							91	M

Nevertheless, a numerical evaluation using the weighted mean method of the parameters OHIP49 (Oral Health Impact Profile), CPQ11-14 (Child Perceptions Questionnaire), and patient satisfaction was performed. The results are displayed in Table 9. The mean baseline score of the Oral Health Impact Profile 49 in prosthetically not treated adults was 27.8 (CI 0.9) points of a possible maximum of 196 points (14.1 % of maximum) indicating that the patients were not strongly limited by the disease. The baseline scores of the Children Perceptions Questionnaire 11–14 in untreated children with oligodontia was 26.2 (CI 2.2) of 148 maximum score points (17.7 % of maximum). A mean improvement of 14.9 OHIP points after prosthetic treatment and occlusal rehabilitation was calculated from three reporting studies. The reported improvement with conventional prosthetics was 19.5 points (only one study), with implant prosthetics 12.5 score points (only two studies). Patient satisfaction rates after treatment was 66.5 % (one study) with orthodontic space closure 75 % in autotransplants (one study), 76.6 % with conventional prosthetics (two studies), and 93.4 % with implant prosthetics (3 studies) in hypodontia patients (Table 9).

**Table 9:** Numerical results of patient-centered outcome parameters (weighted mean values)

OHIP	Baseline OHIP	95 % confid. interval	OHIP improvement general	95 % confid. interval	OHIP improvement implants	95 % confid. interval	OHIP improvement conventional	95 % confid. interval
[Score units]	27.8	0.9	14.9	1.7	12.5	0.2	19.5	n.a.
CPQ	Baseline CPQ							
[Score units]	26.6	2.2						
Patient satisfaction	Satisfaction orthod. clos		Satisfaction AutoTX		Satisfaction conventional		Satisfaction implants	
[%]	66.5	n.a.	75.0	n.a.	76.6	5.2	93.4	19.3

Two assessments of self-esteem were available. Hashem found self-esteem not significantly affected in hypodontia patients compared to normal control patients. Finnema observed an improvement of patients' self-esteem in 61 % of cases after treatment with dental implants (Table 9).

#### Studies on chewing efficacy

Two studies were retrieved on chewing efficacy data after prosthetic treatment of hypodontia with implants. Both studies used different methods of assessment. In the study of Finnema and coworkers [27], the mandibular function impairment questionnaire (MFIQ) dropped from 2.23 (44.6 % of maximum scoring range of 5) at baseline to 0.31 after occlusal rehabilitation. The study of Goshima and coworkers [28] reported a marked improvement of bite force, masticatory index and functional impairment index, reduced chewing time, and increased occlusal contact area after prosthetic treatment with dental implants (Table 10).

**Table 10:** Synopsis of studies of chewing performance before and after treatment with dental implants

Autor	Year	Study type	Population	Treatment	Comparison	Patients	Replaced teeth	Bite force [N]	Color change chew.gum [a*]	Chewing time [s]	Occlusal contact area [mm^2^]	Masticatory index 0–3	MFIQ	Study quality
Finnema [27]	2005	Retrospect.	Oligodontia	Implant FPD	Before treatment	13	156						2.23	M
					After treatment								0.31	
Goshima [28]	2009	ProspObs	Oligodontia	Implant sing. crown	Before treatment	18	37	1087.3	18.8	21.1	34.3	0.3		L
					After treatment			1383	23.2	17.9	44.9	0		

### Further studies

#### Studies on orthodontic treatments in patients with congenitally missing teeth

The literature search according to the applied criteria revealed four relevant studies on orthodontic therapy. Since orthodontic therapy is in most cases a supportive therapy and no competing therapy orthodontics were not included into the numerical evaluation. Robertsson and Mohlin [29] compared directly in a cross-sectional study of 50 patients with missing lateral incisors, orthodontic space closure with orthodontic gap opening, and a bridge. The patients were slightly more satisfied with the esthetics of the space closure. There were slight differences in general patient satisfaction (see there) in favor of the bridge and no differences in any functional parameters. A remarkable finding in two studies of Uribe and coworkers [30] was that after orthodontic opening of a gap of an agenesis of a lateral incisor, the alveolar ridge lost 1.6 mm ridge width and 0.6 mm ridge height, measured retrospectively in stone models of 31 patients. In their second study [31], the same problem was measured with cone beam CT and a shrinkage of the width of the alveolar of 0.9 mm and no height reduction was measured in 11 patients. Dueled [26] observed in severe hypodontia the patients’ root resorption in 36 % of oligodontia cases, who received adjunctive orthodontic therapy, which was not found in patients without orthodontic therapy. According to the authors, possible explanations for this finding may have been narrower and more cortical alveolar ridges in edentulous sites of tooth agenesis patients and different tooth root morphology (taurodontism).

#### Studies on craniofacial growth

In this group, 3 studies were retrieved according to the search criteria. The two studies of Dellavia and coworkers [18, 19] contained drawings (polar diagrams) but not the underlying numerical data. Their evaluation of facial photographies demonstrated that patients with ectodermal dysplasia had a slightly reduced global facial growth in comparison with normal reference peers, with a delay of about 2 years in mandibular and maxillary peak developments. The cross-sectional study of Johnson and coworkers [32] analyzed lateral cephalograms of 50 ED patients without treatment with dental implants and compared them with 45 ED patients, who had received dental implants. Craniofacial morphology did not differ significantly between implant-treated and non-treated ED patients.

## Discussion

This systematic review of the literature made a few noticeable findings. Dental implants and implant-borne prostheses demonstrated high survival and success data with approximately 30 % higher survival/success figures than conventional prostheses on teeth. However, in children implant and prosthesis, success was 20 % lower and success 40 % lower than in adults and adolescents. The factor severity of hypodontia, syndromal versus non-syndromal tooth defects, size and type of the prosthesis, maxilla versus mandible, were in comparison to the age group of minor significance for the implant prognosis. The two other non-prosthetic treatment options using natural teeth, tooth autotransplants, and preservation of the deciduous teeth had survival rates in the range of dental implants but lower success rates due to a considerable incidence of ankylosis, root resorption, and infraocclusion. Also, patient satisfaction rates were higher for dental implants compared to the other treatment options.

According to the present data, dental implants in patients with congenitally missing teeth have an excellent documented prognosis with survival rates of 95.3 % after a mean follow-up of 4.6 years. The prognosis even rises to 97.4 %, if only adults are considered taking into account the higher failure rates in children as discussed below. These figures are well lined with currently published data on implant prognosis in conventional dental implant patients. For example, a 95.5–96.3 % 5-year survival rate has been published in a recent meta-analysis [33].

The pictures changes slightly in favor of autotransplants and preservation of deciduous teeth when annual failure rates are calculated by dividing the survival/success data through the years of observation. Still, conventional prosthetics have the highest annual failure rates with approximately 5 %, 5 times higher than the annual failure rates after tooth autotransplantation and preservation of natural teeth with approximately 1 %. The annual failure rate is first of all the mathematical result of the 2–3 times longer observation time in the included studies on the treatment options using natural teeth. On the other hand, this finding confirms the clinical experience that a once healed autotransplant or a once preserved deciduous tooth has in contrast to prosthetic components causes hardly any maintenance and repair expenditures. The latter problem is even more evident, if in the present data, the annual survival-based failure rate of 5 % for conventional prosthetics is compared with the annual success-based failure rate of conventional prosthetics of 11 %. Survival means that the prosthetic component is still in the oral cavity whereas success can be much lower due to prosthetic complications and treatment needs. Here, the presented 3 % annual failure rate for dental implants ranges in the middle between conventional prosthetics and treatments with natural teeth. Again, this figure is pretty much in line with recent findings on annual failure rates in implants in conventional dental implant patients. A recently published meta-analysis on implants in conjunction with sinus floor augmentations reported an annual failure rate of 3.5 % [34].

The low prognosis of dental implants in children (72.4 %) compared to adolescents (93.0 %) and adults (97.4 %) was not surprising. Also, the systematic review of Yap and Klineberg [11] came to this conclusion. But the large difference of 25 % was remarkable and is clinically relevant. The high annual failure rates of implants in children according to the included studies of 50.2 % compared to 4.6 % in adolescents and 0.7 % in adults with congenitally missing teeth can be alarming. This is in part again an effect of the lower observation times in the included studies (factor 1.5). At this point, also the inclusion of the observation of Bergendal and coworkers [20] has to be discussed, who observed in a survey in Swedish specialist dental clinics an implant loss rate of 6.1 in adolescents and 64.3 % in children under the age of 13. She reported only healing failures of the implants and no long-term problems in children. The data have been included in this systematic review of the literature here, although it is not a true clinical study and the observation time of 6 months is arbitrary and short. The relevance of the observation of Bergendal has already been discussed elsewhere in the literature [35]. The study was also included because the finding fits to other studies in this review. A biological explanation of healing problems of dental implants in young children may be the brittle cortical bone structure and a more active immune system in children compared to adults and adolescents. An international Delphi consensus group, too, did not reach consensus on the use of dental implants in growing children affected by hypodontia [9, 10]. Decision making for dental implants in children and adolescents cannot only be based on survival data. It includes also secondary infraocclusion of restorations on implants which can account for the upper incisor region up to 2.2mm [3]. Less infraocclusion had been observed for teeth in the lower jaw and upper canines [3].

According to the setting of the second consensus conference of the German Implant Association, in this systematic review, special emphasis had to be laid on patient-reported and patient-centered outcomes. In summary, the retrieved studies showed that patients with hypodontia are less disabled than expected, as demonstrated by the moderate OHIP and CPQ scores. For example, according to a study in edentulous wearers of full dentures before implant stabilization, the baseline OHIP score was much higher (54.2) [36], compared to a mean baseline score in the studies included here of 27.8. This observation may be explained by the adaptation of the juvenile hypodontia patients to the situation from early childhood. Patients do not know it differently. This has also an impact on measurements of self-esteem, which according to Hashem and coworkers was not significantly different between hypodontia patients and control patients [37]. Nevertheless, an effect of occlusal rehabilitation was measurable with quality of life data in three available studies on that topic in the present data. Obviously, there is a lack of clinical studies using quality of life data in the field of congenitally missing teeth. Due to heterogeneity and low number of studies, data with patient-reported outcomes in this review have to be interpreted with caution. The same restrictions apply to interpretations of the presented data on the effect of occlusal rehabilitation on masticatory performance in hypodontia patients.

The PICO question asked whether an early occlusal rehabilitation with dental implants in comparison to tooth autotransplants, conventional prosthetics on teeth, or preservation of deciduous teeth has a better outcome. Based on the presented data here, the question can be answered with yes.

However, each treatment has its time. Preservation of deciduous teeth and autotransplantation is an ideal option in children and adolescents, when dental implants have reduced success rates. The latter option can also be used as a temporary solution until completion of growth. As shown here by the OHIP and self-esteem data and facial growth data, patients with severe oligodontia benefit from early occlusal rehabilitation. A practicable way to safe application of implants in children affected by severe oligodontia may be the proposal by Heuberer and coworkers [38], who used with good success onplants in the maxilla placed in the palate behind the teeth to fix an overdenture prosthesis. In this region, less infraocclusion and less interference with transversal palatal growth are expected. Accordingly for the same reasons, implants can be placed in the mandibular canine region. Also, costs play a role in clinical decision-making with tooth autotransplantation being the most cost-effective option [39] along with preservation of primary teeth, which virtually causes no costs. If the costs are manageable, in clinical decision-making, conventional tooth-borne prosthetic solutions should be thoroughly weighted against implants.

## Conclusion

In synopsis of general and patient-centered outcomes, implants yielded the best results, however, not in children younger than 13 years. Autotransplants and deciduous teeth had low annual failure rates and are appropriate treatments in children and adolescents at low costs. Conventional prosthetics had lower survival/success rates than the other options. Due to heterogeneity and low number of studies, patientreported outcomes in this review have to be interpreted with caution.
